# Impact of plateau pika burrowing activity on the grass/sedge ratio in alpine sedge meadows in China

**DOI:** 10.3389/fpls.2022.1036438

**Published:** 2022-12-30

**Authors:** Xiang Yao, Haoran Wang, Saiqi Zhang, Maria Oosthuizen, Yilin Huang, Wanrong Wei

**Affiliations:** ^1^ Jiangsu Key Laboratory for the Research and Utilization of Plant Resources, Institute of Botany, Jiangsu Province and Chinese Academy of Sciences (Nanjing Botanical Garden Mem. Sun Yat-Sen), Nanjing, China; ^2^ Sichuan Jiuma Expressway Co. Ltd., Aba, China; ^3^ Department of Zoology and Entomology, University of Pretoria, Hatfield, South Africa; ^4^ Key Laboratory of Southwest China Wildlife Resources Conservation (Ministry of Education), College of Life Sciences, China West Normal University, Nanchong, China

**Keywords:** bare soil, grass, sedge, graminoid, pasture yield, plateau pika

## Abstract

**Introduction:**

Burrowing activities of plateau pikas cause widespread bare patches in alpine meadows on the Qinghai-Tibet Plateau, affecting the plant community composition and forage production. However, it is not clear how these bare patches influence the main forage composition in alpine meadows.

**Methods:**

Therefore, we investigated the plant communities in bare patches (BP) and neighboring control plots (CK) in alpine meadows in Maqu county in the Gannan region of China.

**Results:**

Our results showed that plant communities in the CK plots differed from those in the BP plots. The sedge cover, number of sedge species and number of grass species were all significantly higher in the CK plots compared to the BP plots. However, grass cover and its dry weight were significantly higher in the BP plots. Grass cover and the grass dry weight in the BP plots were 1.859 times and 1.802 times higher than that in the CK plots across the five sites, respectively. Grasses also had a significantly higher cover and dry weight than sedge in the BP plots, grass cover was 66.5 times higher than the sedge cover, and the grass dry weight was 68.242 times that of the sedge dry weight. Therefore, bare patches resulting from plateau pika burrowing activity significantly increase the grass/sedge ratio in alpine meadows.

**Discussion:**

A potential explanation is that grasses have a stronger reproductive potential than sedges in bare soil. This has implications for pasture yields since grasses have a higher biomass per unit area compared to sedges in alpine meadows.

## Introduction

The Qinghai-Tibet Plateau (QTP) plays important ecological and economic roles such as climate regulation, supporting wild animals and plants and animal husbandry (Kang et al., 2007). It also serves as the source region of the two most important rivers in China, the Yangtze and Yellow rivers, and is therefore very important for the conservation of water and soil (Sun et al., 2017). The habitat of the QTP consists primarily of alpine meadows and alpine grasslands. Alpine meadows are at higher elevations, between 3200 and 4800 m on the QTP in China (Qiao and Duan, 2016), and are dominated by sedges (*Kobresia* spp., Cyperaceae) accompanied by grasses and other forbs. At lower elevations, alpine grasslands are dominated by grasses (Gramineae) that are accompanied by forbs (He and Richards, 2015; Lu et al., 2015). Both sedges and grasses are high quality forage for livestock in alpine meadows, but these plant types have different traits. Grasses have a higher plant height and higher biomass per unit area compared to sedges in alpine meadows (Dong et al., 2007). Hence, the sedge/grass proportion will determine the pasture yield. Therefore, it is important to be mindful of the forage composition in alpine meadows.

Recent literature yielded a number of studies regarding the changes in plant community composition in alpine meadows. The plant community in alpine meadows is sensitive to changes in environmental factors such as grazing intensity, climate change, invasive plants, the refuge effect of unpalatable plants and rangeland degradation (Liu et al., 2018). Even light grazing of livestock (Yang et al., 2016) and grazing during the growth period of grasslands (Wei et al., 2022a) can increase plant species richness, while heavy grazing increases the proportion of unpalatable plants and decreases the proportion of palatable graminoids, the plant cover and biomass (Yang et al., 2016; Ji et al., 2020). The plant community composition and biomass are also highly sensitive to climate change in alpine meadows (Yang et al., 2011). For example, higher temperatures increase plant biomass but decreases plant diversity (Baldwin et al., 2014). The proportion of grasses increased while sedges and forbs decreased in alpine meadows following climate warming on the Qinghai-Tibet Plateau (Liu et al., 2018). In addition, another study showed that experimental warming altered the graminoid composition by increasing the frequency of sedges but reducing the frequency of grasses in a subalpine meadow of the Rocky Mountains, USA (Rudgers et al., 2014). However, herbivores can mitigate the response of plant communities to climate change through selective foraging (Olofsson et al., 2004; Post and Pedersen, 2008). Invasive plants primarily influence plant community composition due to their high resource-use efficiency, toxicity or other traits (Funk, 2013). The refuge effect of unpalatable plants increases the plant diversity (Yao et al., 2015), the graminoids diversity and the proportion of grass in alpine sedge meadows (Yao et al., 2019). Moreover, alpine meadows are widely degraded, and the level of degradation has different effects on the cover and biomass of different plant functional types such as sedges, grasses, legumes and forbs (Li et al., 2015).

Rodent activity is also known to regulate vegetation structure. For example, disturbances from prairie dogs (*Cynomys ludovicianus*) maintain grassland and savanna by preventing the establishment of woody species (Weltzin et al., 1997), while pocket gophers (*Geomys bursarius*) regulate the vegetation structure by facilitating seedling establishment in alpine meadows (Forbis, 2003). Plateau zokors (*Myospalax fontanierii*) reduce vegetation cover and height in alpine meadows (Zhang and Liu, 2003). As a habitat, alpine meadows support a large number of plateau pikas (*Ochotona curzoniae*). Pikas are some of the most representative wild animals in alpine meadows (Qu et al., 2013; Qu et al., 2019), and impact the plant community composition and forage production with their burrowing and feeding activities (Dorji et al., 2014; Zhang et al., 2020; Wei et al., 2022b).

Pika activities can have both positive and negative effects on alpine meadows. Plant species richness is higher in the patches where plateau pikas burrowed compared to control areas in the high-altitude arid rangelands of Trans-Himalaya (Bagchi et al., 2006), however, in bare patches following plateau pika burrowing activities, plant species richness was reported to be significantly lower than in neighboring undisturbed areas (Wei et al., 2007). Intermediate disturbance levels from plateau pika burrowing activities can be beneficial by increasing the forage quality and plant diversity in the alpine meadows (Guo et al., 2012; Liu et al., 2017; Pang and Guo, 2018). Plateau pika burrowing disturbances also benefit clonal growth of sedges (Wang et al., 2020) and the biomass of sedges in alpine meadows is positively correlated with increased burrow density (Liu et al., 2017). Pikas (*O. princeps*) are regarded as allogenic engineers, and increase the nutrient availability of soil to plants in North America (Aho et al., 1998). High-density plateau pika grazing decreases the biomass of palatable plants in alpine meadows (McIntire and Hik, 2005; Guo et al., 2012; Sun et al., 2015; Liu et al., 2017; Zhang et al., 2020), but increases the biomass and species richness of the other forbs (Zhang et al., 2020). The effects of plateau pikas on alpine meadows appear to be density dependent, since at low densities, plateau pikas have no significant effect on the plant species richness or diversity on winter pastures in alpine meadows (Wei et al., 2020a). The impact of plateau pikas on alpine grassland vegetation is also not uniform within the home range of pika families (Wei et al., 2019). The above examples illustrate the complex nature of plant communities and their interactions with biotic and abiotic factors. However, to date, it is not known how bare patches created by plateau pika burrowing activities influence the grass/sedge ratio in alpine meadows.

We predict that bare patches resulting from plateau pika burrowing will have an inconsistent influence on the biomass and coverage of sedges and grasses, which may change the sedge/grass ratio in alpine meadows. We conducted a study in a representative alpine meadow in Maqu county on the Gannan plateau of China, with the objective to determine the effect of bare patches from plateau pika burrowing activities on the ground cover and biomass of grasses and sedges.

## Materials and methods

### Study area

This study was performed in Maqu county in the Gannan Tibetan autonomous prefecture located on the north-eastern Qinghai-Tibet Plateau (33° 40′ N, 101° 54′ E, 3430 m). The research was conducted in a summer grazing rangeland dominated by sedges (*Kobresia pygmaea* and *K. kansuensis*) and grasses (*Poa crymophila* and *Elymus nutans*). This region has a short warm season from June to September and a long cool season from October to May. The mean annual temperature is 1.2°C, the mean annual precipitation is 564 mm (Wei et al., 2019). The Plateau pika is the most prominent small mammal herbivore that is supported by the alpine meadows.

### Study design

**Figure 1:**
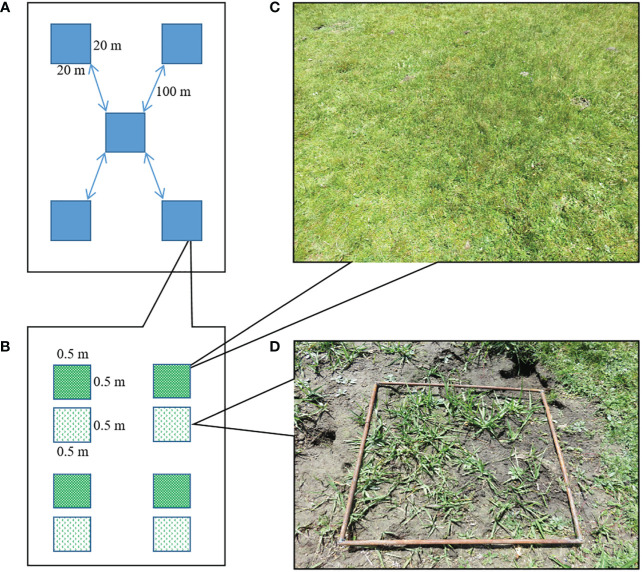
Study design. **(A)** Five sites were selected, **(B)** each site had two types of plots, **(C)** a control plot, **(D)** and a bare patch plot created by plateau pika burrowing activity.

In order to investigate the effect of bare patches from plateau pika burrowing activity on the plant community, specifically graminoid species (grasses and sedges), therefore bare patches were selected on the condition that graminoid species occur on it. Five sites were selected using the five-point sampling method (**Figure 1A**) and the sites were approximately 100 m apart. Each site consisted of a 20 m x 20 m square and contained four replicates of a 0.5 m x 0.5 m control plot (CK) and a 0.5 m x 0.5 m bare patch plot (BP) (**Figure 1B**). Control plots (**Figure 1C**) and experimental plots (**Figure 1D**) were in close proximity (less than 1m) to avoid environmental differences of habitat such as edaphic and climatic conditions. Our survey showed that the active burrows density at the five sites was between 800 and 900 burrows per ha, which falls in the high burrow density range according to Wei et al. (2020b).

### Plant community investigation

Plant cover, height and aboveground biomass for each species were determined in August 2019. These indices were then compared for each plant species in the CK plots and the BP plots. Our study focused on differences in graminoid species (in particular Poaceae and Cyperaceae) in the different plots, because they are dominant plant species and important forage for livestock (yak and sheep) in alpine meadows. Livestock had free access to all the plots. The plant cover and plant height of each species were recorded in situ. Plant cover was measured by using the point frame method (Godínez-Alvarez et al., 2009). Plant height was calculated by the average height of 10 randomly selected individual plants per species per plot. All plant species were then clipped to ground level for dry weight measurement. The dry weight of each plant was measured in the laboratory after being oven-dried at 75°C for 48 h.

### Statistical analysis

Principal coordinates analysis (PCoA) was applied to visualize the difference of plant community composition between the CK plots and the BP plots. For PCoA analysis, the plant cover of each plant species was used. Diversity was calculated with the Shannon–Wiener index (Spellerberg and Fedor, 2003).

Data analysis was performed with IBM SPSS 25.0. A nonparametric, Mann-Whitney U test was used to test statistical significance across the whole data set, because the data of cover, plant height and aboveground biomass of some species were not normally distributed. The effect of bare patches from pika burrowing activity on plant communities was assessed using a mixed-effect ANOVA by treating “study site” as a random effect (Verbeke and Lesaffre, 1996). The statistical significance was defined at the 95% confidence level (P = 0.05).

## Results

### Effect of bare patches on the plant community similarity

**Figure 2:**
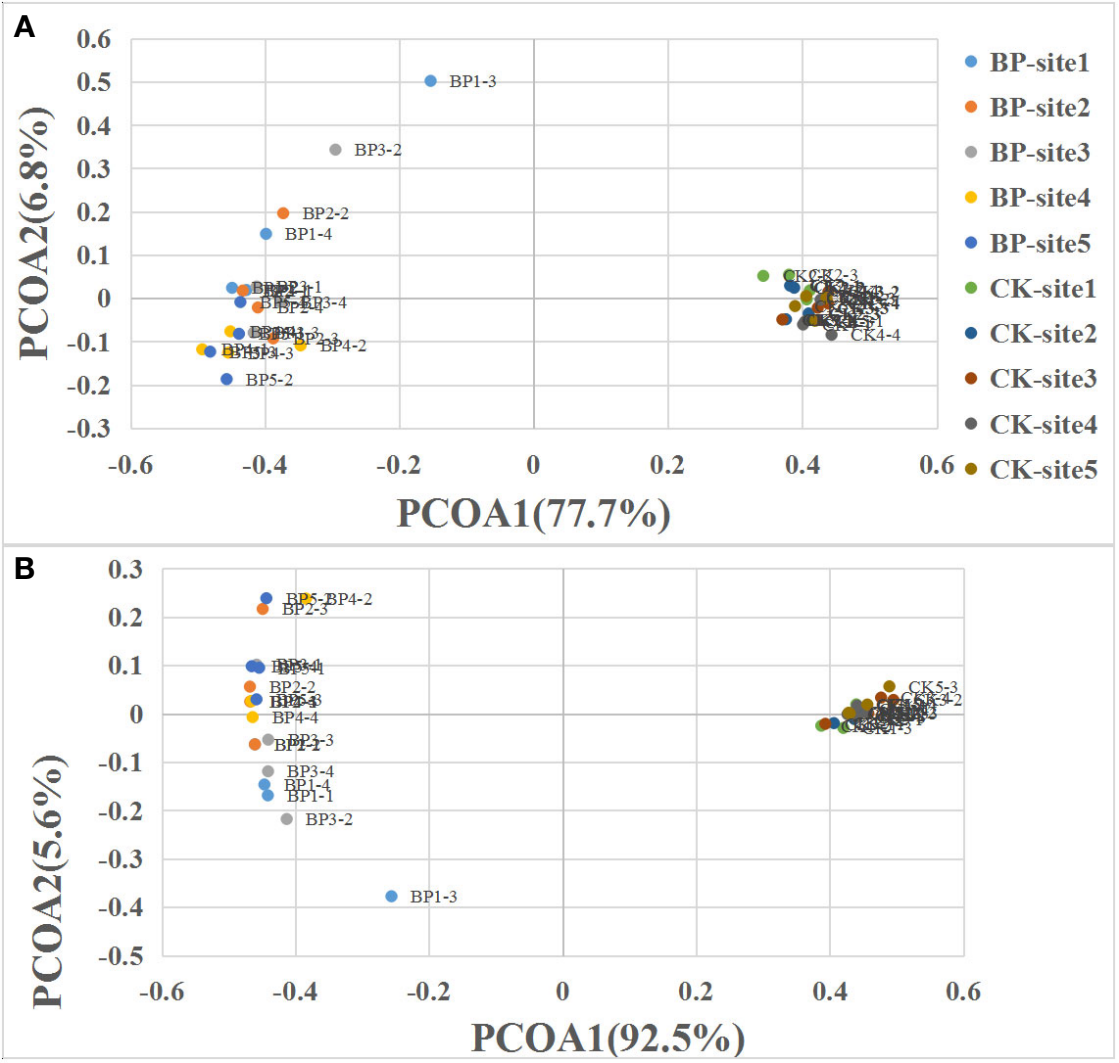
Principal coordinates analysis (PCoA) of **(A)** the plant community and **(B)** the graminoid community. Graminoids included grasses and sedges.

In the control plots, the most abundant plant species was *Kobresia humilis*, *K. kansuensis*, *Elymus nutans*, *Poa crymophila* and *Potentilla fragarioides*, whereas the five most abundant plant species in the bare patch plots were *E. nutans*, *P. anserina*, *Saussurea japonica*, *Medicago falcata* and *Lancea tibetica*. The complete plant species list and their cover are presented in **Supplementary Table S1**. Based on the PCoA analysis, it was clear that plant communities in the CK plots were different from plant communities in the BP plots (**Figure 2A**). Plant communities in the CK plots were highly similar in all the five sites while plant community in the BP plots had more variation (**Figure 2A**). Graminoid communities also showed high similarity in CK plots while there was more variation in the BP plots (**Figure 2B**). PCoA 1 explained 77.7% of the variation in plant communities (**Figure 2A**) and 92.5% of the variation in graminoid communities (**Figure 2B**).

### Plant community differences in the bare patch plots and the control plots

Plant communities had a significantly higher total cover (Z= -2.611, *P* =0.009), total dry weight (Z= -2.611, *P* =0.009) and a higher plant diversity (Z= -2.611, *P* =0.009) across the five sites in the control (CK) plots compared to the bare patch (BP) plots (**Table 1**). There was a significantly higher cover of sedge (Z= -2.611, *P* =0.009), a higher number of sedge species (Z= -2.668, *P* =0.008) and higher number of grass species (Z= -2.835, *P* =0.005) in the CK plots than in the BP plots across the five sites (**Table 1**). There was a significantly higher cover (Z= -2.402, *P* =0.016) and dry weight of grass (Z= -2.402, *P* =0.016) (mainly *Elymus nutans* in this study) in the BP plots compared to the CK plots across the five sites (**Table 1**). The grass cover and grass dry weight in the BP plots were 1.859 times and 1.802 times higher than in the CK plots across the five sites (**Table 1**), respectively. The mixed-effect ANOVA revealed that bare patches significantly influenced the above-mentioned indices in all the five sites (**Table 2**).

**Table 1:** Mean value of plant indices across the five sites.

	Mean_CK_ ± SE	Mean_BP_ ± SE	Z	*P*	Mean_CK_/Mean_BP_
**Total cover (%)**	99.055^*^ ± 0.155	49.051 ± 2.778	-2.611	0.009	2.019/1
**Total dry weight (g/0.25m^2^)**	31.017^*^ ± 1.010	15.970 ± 1.008	-2.611	0.009	1.942/1
**Plant diversity**	2.258^*^ ± 0.037	1.135 ± 0.075	-2.611	0.009	1.989/1
**Grass cover (%)**	16.100 ± 1.407	29.925^*^ ± 3.170	-2.402	0.016	**1/1.859**
**Sedge cover (%)**	61.381^*^ ± 2.211	0.450 ± 0.189	-2.611	0.009	136.402/1
**Grass species number**	3.000^*^ ± 0.000	1.100 ± 0.061	-2.835	0.005	2.727/1
**Sedge species number**	2.200^*^ ± 0.122	0.750 ± 0.209	-2.668	0.008	2.933/1
**Grass dry weight (%)**	5.642 ± 0.659	10.168^*^ ± 1.035	-2.402	0.016	**1/1.802**
**Sedge dry weight (%)**	20.084^*^ ± 1.168	0.149 ± 0.073	-2.611	0.009	134.792/1

Mean_CK_, mean value in the CK plot, Mean_BP_, mean value in the BP plot. Significant differences of mean values between in the CK plot and the BP plot are indicated: ^*^P < 0.05. Bold value means this result is emphasized.

**Table 2:** Effects of bare patches resulting from plateau pika burrowing activity and sites on plant community as analysed by a mixed-effect ANOVA.

		Bare patch	Site
	*d.f.*	1	4
**Total cover**	*F*	69.691	0.351
	*P*	**0.000**	0.842
**Total dry weight**	*F*	51.127	0.706
	*P*	**0.000**	0.594
**Plant diversity**	*F*	175.513	1.317
	*P*	**0.000**	0.283
**Grass cover**	*F*	40.337	2.26
	*P*	**0.000**	0.083
**Sedge cover**	*F*	3050.844	1.548
	*P*	**0.000**	0.211
**Grass species number**	*F*	619.098	0.773
	*P*	**0.000**	0.551
**Sedge species number**	*F*	92.367	3.432
	*P*	**0.000**	**0.018**
**Grass dry weight**	*F*	31.206	2.747
	*P*	**0.000**	**0.044**
**Sedge dry weight**	*F*	3705.335	3.517
	*P*	**0.000**	**0.017**

Bold value means P < 0.05.

Sedges had a significantly higher cover (Z= -2.611, *P* =0.009) and higher dry weight (Z= -2.611, *P* =0.009) than grasses in the CK plots in all the five sites (**Table 1**). In the CK plot, the sedge cover/grass cover ratio was 3.812, and the sedge dry weight/grass dry weight ratio was 3.560. Grasses had significantly higher cover (Z= -2.611, *P* =0.009) and a higher dry weight (Z= -2.611, *P* =0.009) than sedges in the BP plots in all the five sites. In the BP plot, the grass cover/sedge cover ratio was 66.5, and the grass dry weight/sedge dry weight ratio was 68.242.

## Discussion

There are several factors that affect the plant composition in alpine meadows, including grazing density (Yang et al., 2016; Ji et al., 2020), temperature and precipitation (Yang et al., 2011; Baldwin et al., 2014), invasive plants (Funk, 2013), refuge effect of unpalatable plants (Yao et al., 2015; Yao et al., 2019), and burrowing activities of rodents (Dorji et al., 2014; Pang and Guo, 2018; Wang et al., 2020). However, it is not clear how bare patches resulting from plateau pika burrowing activities influence the grass/sedge ratio in alpine meadows. The plateau pika is one of the most widely distributed small animals in alpine meadows. Their continuous burrow digging activity during the day and night has earned them the title of ecosystem engineers (Smith and Foggin, 1999; Lai and Smith, 2003; Wei et al., 2019), that has a large effect on the vegetation structure. In this study, we investigated the plant communities in and around bare patches created by plateau pikas with their burrowing activities in alpine sedge meadows, and found an increased grass:sedge ratio in the bare patches.

### Plant community differences between in the bare patch plots and the control plots

Plant communities are more similar in the CK plots than in the BP plots. This is likely because plant communities in the CK plots have been stable for many years while the BP plots are disturbed and modified to open habitats available for plant colonization. The plant communities in the BP plots are largely influenced by factors such as herbivore grazing and the native plant community (Shang and Long, 2007). Similar to our results, a previous study reported that control plots are not significantly different from each other, but variance in the bare patch plots is approximately twice that of the control plots, and can be attributed to the lower species richness in bare patch plots (Smith et al., 2019). In addition, we showed that plant communities have a higher total cover and total dry weight in the CK plots compared to the BP plots. That is probably because newly colonized plants have a lower underground biomass or alternatively, that bare soil is less fertile. Furthermore, alpine meadows have a low mean annual temperature (1.2°C) that is not optimal for plant growth, therefore it is difficult for plants to get established within one or two years in the BP plots.

### Grasses have stronger sexual reproduction than sedges in bare soil

Previous research indicated that the biomass of sedges increases while the biomass of grasses initially increases and then decreases with increasing burrowing density (Guo et al., 2012; Liu et al., 2017). Moderate disturbances by plateau pikas increases the proportion of graminoids, including grasses and sedges in meadows by increasing graminoid bud density, while a high level of disturbance deteriorates alpine meadows by reducing the graminoid bud density (Wang et al., 2018). In addition, disturbance by plateau pikas benefits clonal growth of sedges in alpine meadows (Wang et al., 2020). In this study, we found that the BP plots have significantly lower sedge cover, a lower number of sedge species and a lower number of grass species than the CK plots, and grasses have higher cover and more biomass than sedges in the BP plots. This indicates that bare patches resulting from plateau pika burrowing initially show an increase in grasses and not sedges. Consistent with our results, a previous study showed that grasses increase while sedges decrease in alpine meadows following climate warming on the Qinghai-Tibet Plateau (Liu et al., 2018). That is possibly because grasses have stronger sexual reproduction than sedges, and can colonize the bare soil more easily and faster. For example, no seedlings of the sedge *K. pyagmaea* were found on exposed soil in the control experiment and no seedlings of *K. myosuroides* were recorded in alpine meadows (Forbis, 2003), while *Kobresia humilis* seedlings were occasionally observed in alpine meadows (Deng et al., 2001). In contrast, the germination rate of *Elymus* spp. exceeds 80% in alpine meadows on the Qinghai-Tibet Plateau (Bu et al., 2008). This suggests that grasses can colonize bare soil resulting from plateau pika burrowing much faster and with more ease than sedges. Besides, there could also be additional factors that affect the grass/sedge ratio in this study, for example a change in soil properties and change of soil seed banks of bare patches. These factors could be included in future research. A previous study reported that toxic bunchgrass (*Achnatherum inebrians*) was beneficial for seed production of grasses i.e. sexual reproduction in grazing alpine sedge meadows since it deters livestock (Yao et al., 2019). This promotes the increase of grasses in the alpine sedge meadows.

### Effect of bare patches from plateau pika burrowing on the grass/sedge ratio

The plateau pika is one of the most representative wild animals that inhabit the alpine meadows (Wang et al., 2020). In recent years, their densities increased as the meadows degraded. Bare soil resulting from plateau pika burrowing activities accounts for 62.6% of the degraded rangeland in the Qinghai province, and for 25% of the degraded rangeland on the Qinghai-Tibet Plateau (Dong et al., 2013). High densities of plateau pikas will increase the effect on the grass/sedge ratio in alpine meadows.

Grasses and sedges are the most important forages for herbivores, but they have different plant traits. Grasses (*Elymus nutans*) have a higher plant height and more biomass than *Kobresia* spp. (Dong et al., 2007), and are widely distributed, from low to very high elevations (1000-5000 m) (Lu, 1993). *Kobresia* spp. plants are generally distributed at higher elevations from 3000 m to 5960 m (Miehe et al., 2008; Wu et al., 2011). In recent years, the climate is warming, causing the proportion of grasses to increase in alpine meadows on the Qinghai-Tibet Plateau (Liu et al., 2018). Grasses may be able to adapt better to environmental changes, and the increased grass proportion in alpine meadows may render a higher pasture yield.

## Conclusion

Plant communities in the CK plots were different from plant communities in the BP plots. There was a significantly higher cover of sedge in the CK plots compared to the BP plots. However, there was significantly higher grass cover in the BP plots than in the CK plots. Grass had significantly higher cover and higher dry weight than sedge in the BP plots. Therefore, we suggest that the bare patches resulting from plateau pika burrowing activities significantly increased the grass/sedge ratio in alpine meadows. That is possibly because grasses have stronger sexual reproduction than sedges in bare soil, promoting the fast colonization of grasses in disturbed and modified habitats. Hence, the pasture yield may be increased since grasses have more biomass per unit area than sedges in alpine meadows.

## Data availability statement

The original contributions presented in the study are included in the article/**Supplementary Material**. Further inquiries can be directed to the corresponding author.

## Author contributions

XY Wrote the original draft. XY and WW performed the experiment. MO, WW, HW, SZ and YH reviewed and edited the article. All authors contributed to the article and approved the submitted version.
