# Supplementary material for: Impact of plateau pika burrowing activity on the grass/sedge ratio in alpine sedge meadows in China
Source: Front Plant Sci. 2022 Dec 30;13:1036438. doi: 10.3389/fpls.2022.1036438 (PMC9838571; doi:10.3389/fpls.2022.1036438)
Supplement: Supplementary file 1 [file Table_1.doc]

**Impact of plateau pika burrowing activity on the grass/sedge ratio in alpine sedge meadows in** **China**

**Xiang Yao****^1^, Haoran Wang^1^, Saiqi Zhang^2^,** **Maria K. Oosthuizen^3^, Yilin Huang^2^, Wanrong Wei^4^**^*^

^1^Jiangsu Key Laboratory for the Research and Utilization of Plant Resources; Institute of Botany, Jiangsu Province and Chinese Academy of Sciences (Nanjing Botanical Garden Mem. Sun Yat-Sen), Nanjing, China, 210014.

^2^ Sichuan Jiuma Expressway CO LTD, Aba, China, 624000.

^3^Department of Zoology and Entomology, University of Pretoria, Hatfield, South Africa

^4^ Key Laboratory of Southwest China Wildlife Resources Conservation (Ministry of Education), College of Life Sciences, China West Normal University, Nanchong, China, 637002.

^*^Corresponding author: Wanrong Wei. Email: weiwr18@126.com

Supplementary

**Table S1 Cover of each plant species across the five sites**

|  | Mean_BP_±SE | Mean_CK_±SE |
| --- | --- | --- |
| Allium sikkimense | 0.000±0.000 | 0.035±0.024 |
| Anemone coelestina | 0.000±0.000 | 0.500±0.500 |
| Anemone obtusiloba | 0.055±0.055 | 0.875±0.385 |
| Anemone rivularis | 0.000±0.000 | 0.605±0.513 |
| Bistorta vivipara | 0.250±0.250 | 0.000±0.000 |
| Carex moorcroftii | 0.000±0.000 | 0.230±0.142 |
| Delphinium caeruleum | 0.010±0.010 | 0.000±0.000 |
| Elsholtzia densa | 0.075±0.075 | 0.000±0.000 |
| Elymus nutans | 29.725±3.291 | 4.800±0.233 |
| Euphorbia esula | 0.000±0.000 | 0.140±0.140 |
| Gentiana macrophylla | 0.650±0.367 | 1.015±0.212 |
| Gentiana scabra | 0.000±0.000 | 0.010±0.010 |
| Geranium wilfordii | 0.015±0.010 | 0.105±0.053 |
| Gueldenstaedtia verna | 0.070±0.070 | 0.770±0.253 |
| Kobresia humilis | 0.370±0.203 | 53.650±1.312 |
| Kobresia kansuensis | 0.080±0.048 | 7.500±0.946 |
| Koeleria macrantha | 0.200±0.170 | 3.125±0.331 |
| Lancea tibetica | 5.055±2.642 | 1.965±0.369 |
| Leontopodium nanum | 0.000±0.000 | 2.325±0.756 |
| Ligularia virgaurea | 0.010±0.010 | 0.000±0.000 |
| Medicago falcata | 0.660±0.321 | 2.280±1.076 |
| Plantago asiatica | 0.215±0.151 | 0.670±0.134 |
| Poa crymophila | 0.000±0.000 | 8.175±0.993 |
| Potentilla anserina | 7.095±3.426 | 2.175±0.415 |
| Potentilla fragarioides | 0.005±0.005 | 3.875±0.625 |
| Saussurea japonica | 4.415±1.850 | 2.200±0.236 |
| Swertia mussotii | 0.000±0.000 | 0.855±0.176 |
| Taraxacum mongolicum | 0.095±0.073 | 1.150±0.338 |

Note: Mean_CK_ = mean value in the CK plot, Mean_BP_ = mean value in the BP plot.
